# Supplementary material for: CD24 Is a Prognostic Marker for Multiple Myeloma Progression and Survival
Source: J Clin Med. 2022 May 20;11(10):2913. doi: 10.3390/jcm11102913 (PMC9144978; doi:10.3390/jcm11102913)
Supplement: Supplementary file 1 [file jcm-11-02913-s001.zip › jcm-1707030-supplementary.pdf]

Supplementary material

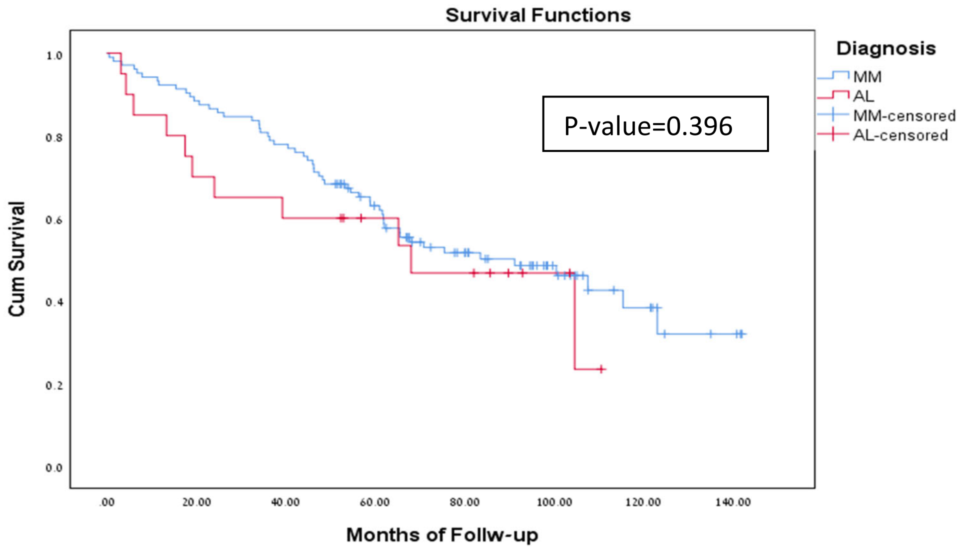

Figure S1: Correlation between OS in MM and AL

Abbreviations: OS: overall survival, AL: Amyloidosis, MM: Multiple Myeloma.

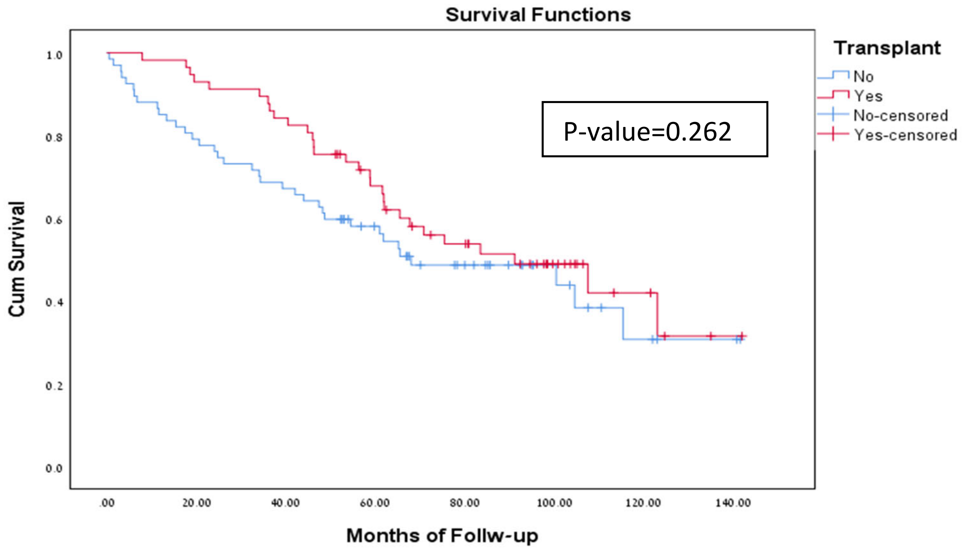

Figure S2: Correlation between OS and ASCT

Abbreviations: OS: overall survival, ASCT: Autologous stem cell transplantation.

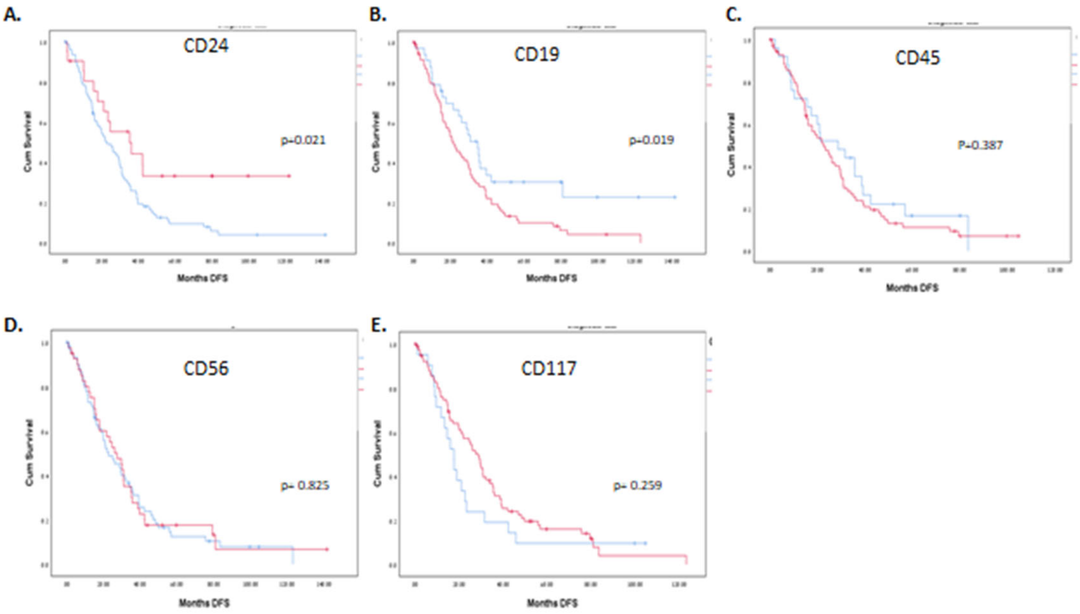

Figure S3: PFS in MM in correlation to CD levels measured by MFC at diagnosis, with a cutoff expression level of 5% on PCs *.

*Red above 5% of PCs, blue below 5% PCs.

Abbreviations: PFS: Progression free survival, MM: Multiple Myeloma

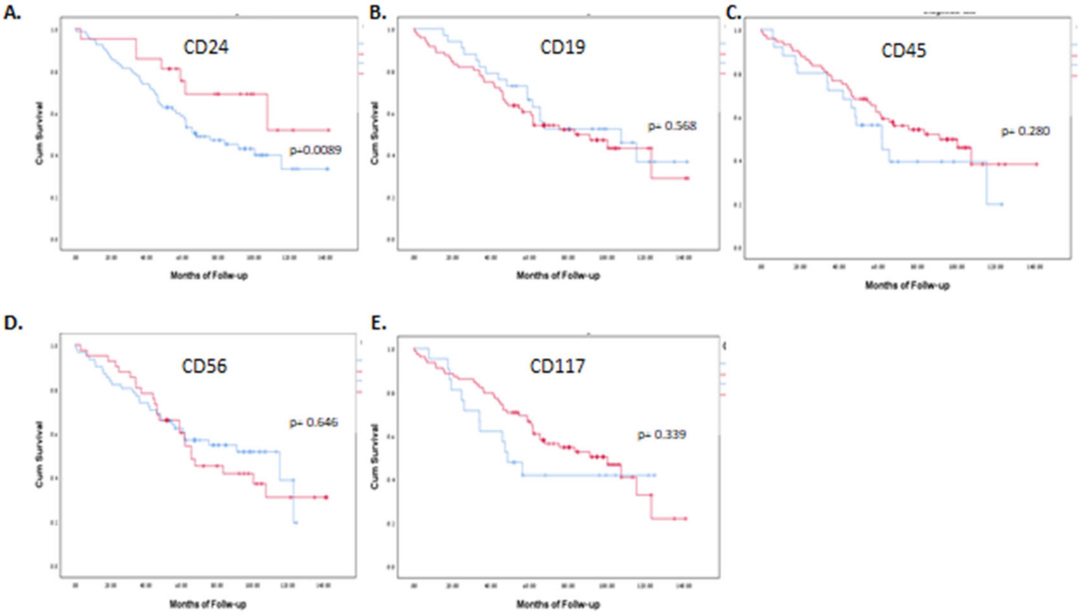

Figure S4: OS in MM in correlation to CD levels measured by MFC at diagnosis, with a cutoff expression level of 5% on PCs * .

*Red above 5% of PCs, blue below 5% PCs.

Abbreviations: OS: Overall survival, MM: Multiple Myeloma

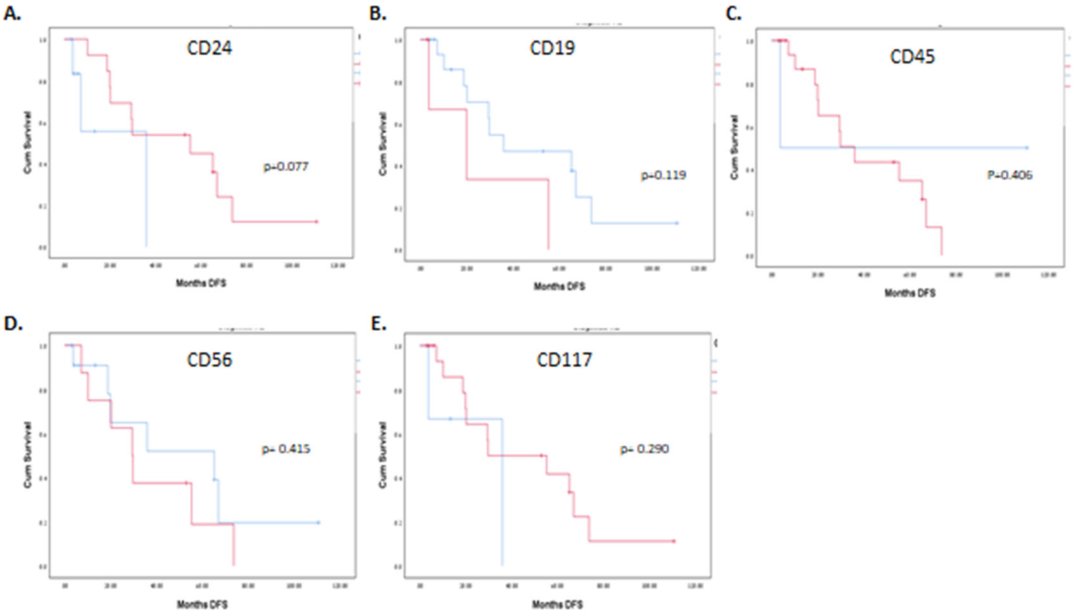

Figure S5: PFS in AL in correlation to CD levels measured by MFC at diagnosis, with a cutoff expression level of 5% on PCs*.

*Red above 5% of PCs, blue below 5% PCs.

Abbreviations: PFS: Progression free survival, AL: Amyloidosis

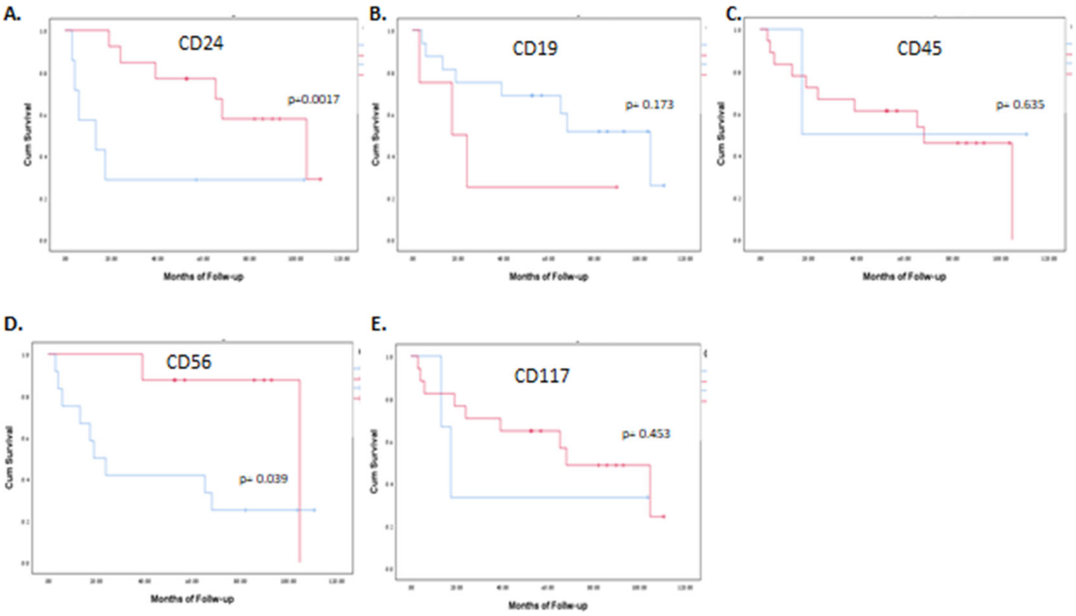

Figure S6: OS in AL in correlation to CD levels measured by MFC at diagnosis, with a cutoff expression level of 5% on PCs*.

*Red above 5% of PCs, blue below 5% PCs.

Abbreviations: OS: Overall survival, AL: Amyloidosis.

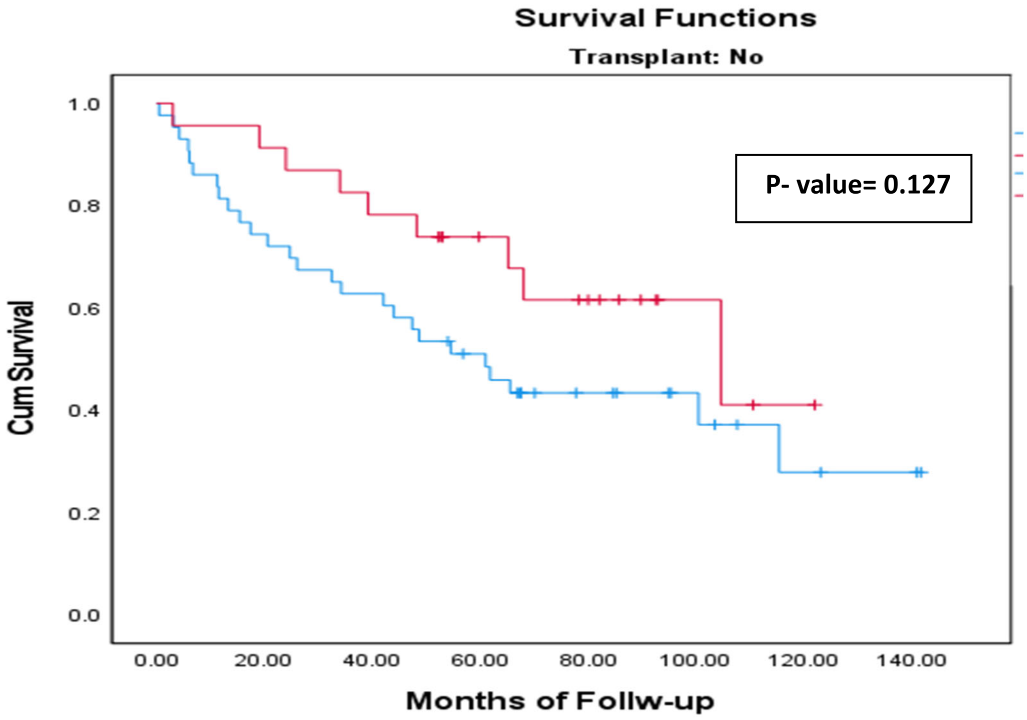

Figure S7: OS in patients who did not went through ASCT in correlation to CD 24 levels measured by MFC at diagnosis, with a cutoff expression level of 5% on PCs*.

*Red above 5% of PCs, blue below 5% PCs.

Abbreviations: OS: Overall survival.

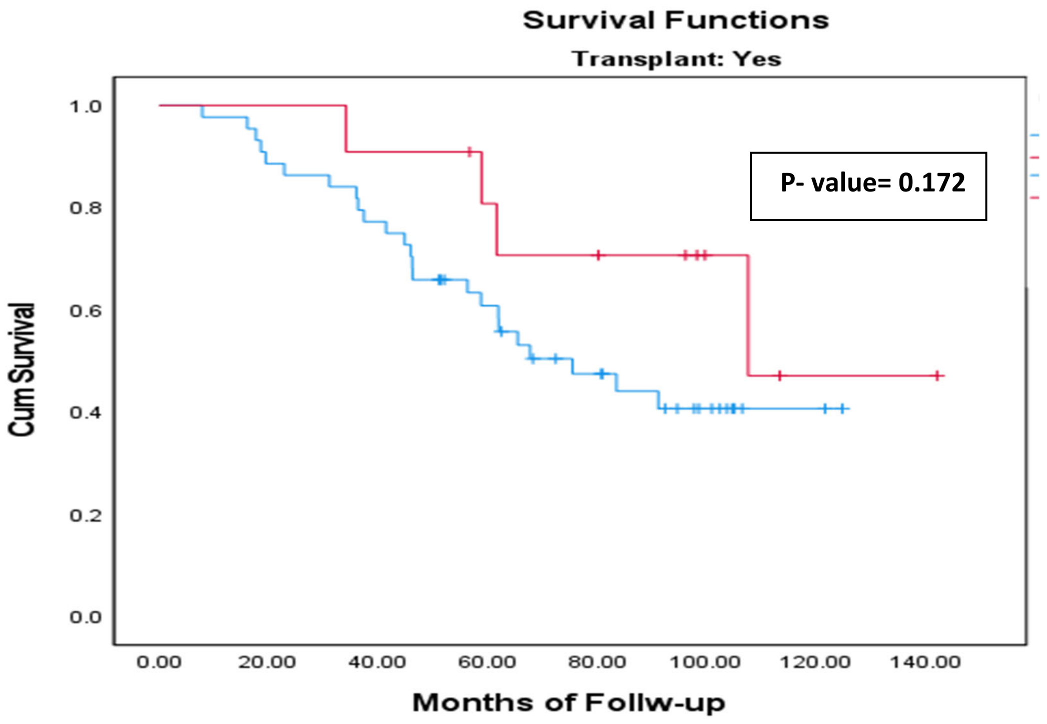

Figure S8: OS in patients who went through ASCT in correlation to CD 24 levels measured by MFC at diagnosis, with a cutoff expression level of 5% on PCs*.

*Red above 5% of PCs, blue below 5% PCs.

Abbreviations: OS: Overall survival.

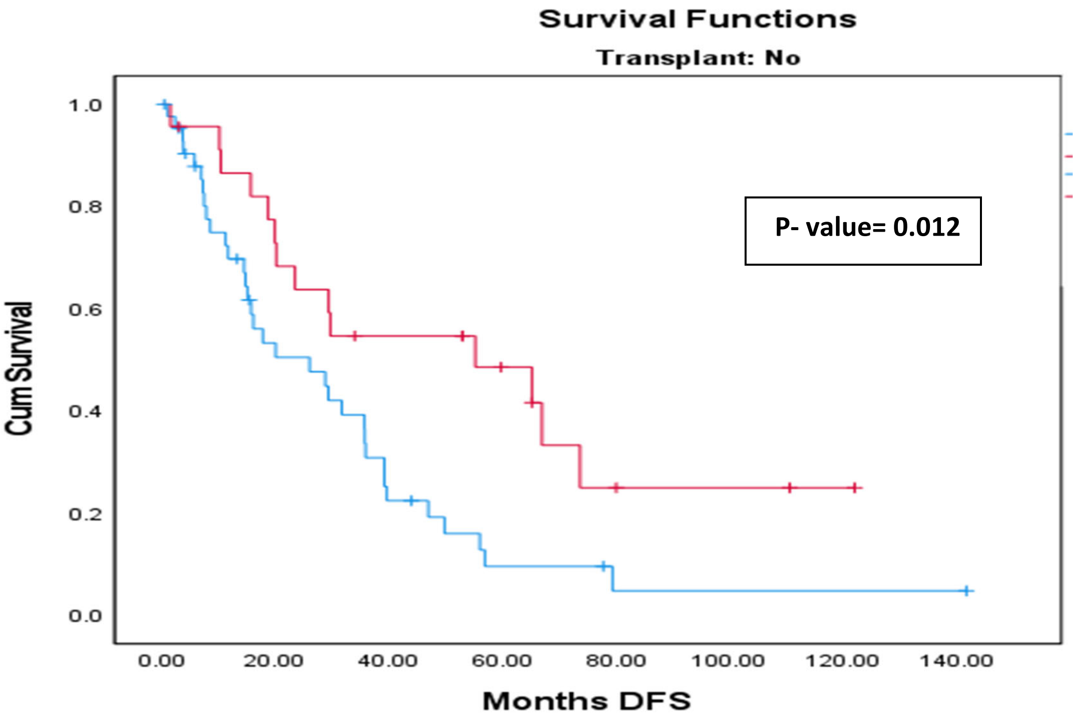

Figure S9: PFS in patients who went through ASCT in correlation to CD 24 levels measured by MFC at diagnosis, with a cutoff expression level of 5% on PCs*.

*Red above 5% of PCs, blue below 5% PCs.

Abbreviations: PFS: Progression free survival.

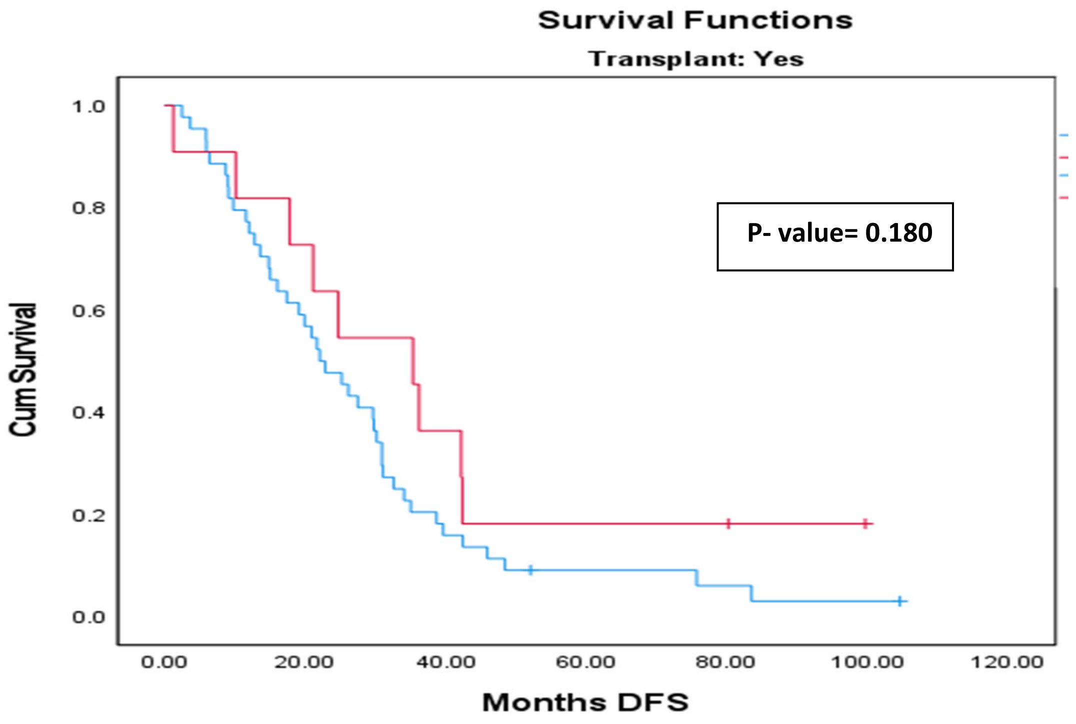

Figure S10: PFS in patients who went through ASCT in correlation to CD 24 levels measured by MFC at diagnosis, with a cutoff expression level of 5% on PCs*.

*Red above 5% of PCs, blue below 5% PCs

Abbreviations: PFS: Progression free survival.
